# FGF, Insulin, and SMAD Signaling Cooperate for Avian Primordial Germ Cell Self-Renewal

**DOI:** 10.1016/j.stemcr.2015.10.008

**Published:** 2015-11-19

**Authors:** Jemima Whyte, James D. Glover, Mark Woodcock, Joanna Brzeszczynska, Lorna Taylor, Adrian Sherman, Pete Kaiser, Michael J. McGrew

**Affiliations:** 1 The Roslin Institute and Royal Dick School of Veterinary Studies, University of Edinburgh, Easter Bush Campus, Midlothian EH25 9RG, UK

## Abstract

Precise self-renewal of the germ cell lineage is fundamental to fertility and reproductive success. The early precursors for the germ lineage, primordial germ cells (PGCs), survive and proliferate in several embryonic locations during their migration to the embryonic gonad. By elucidating the active signaling pathways in migratory PGCs in vivo, we were able to create culture conditions that recapitulate this embryonic germ cell environment. In defined medium conditions without feeder cells, the growth factors FGF2, insulin, and Activin A, signaling through their cognate-signaling pathways, were sufficient for self-renewal of germline-competent PGCs. Forced expression of constitutively active MEK1, AKT, and SMAD3 proteins could replace their respective upstream growth factors. Unexpectedly, we found that BMP4 could replace Activin A in non-clonal growth conditions. These defined medium conditions identify the key molecular pathways required for PGC self-renewal and will facilitate efforts in biobanking of chicken genetic resources and genome editing.

## Highlights

• Avian primordial germ cell self-renewal is dependent on FGF2, insulin, and Activin A molecules
• BMP4 can replace Activin A in non-clonal growth conditions
• Defined culture medium conditions will facilitate studies of germ cell self-renewal in other vertebrate species

## Graphical Abstract

**Figure:**
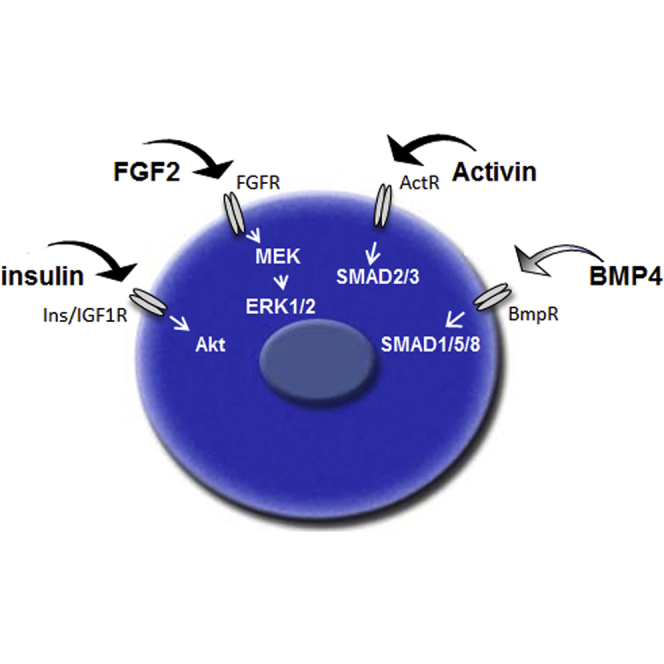

## Teaser

In this article, McGrew and colleagues define the minimal growth factors and signaling pathways needed for the self-renewal of chicken primordial germ cells. They show that self-renewal is dependent on FGF2, insulin, and Activin A growth factors. BMP4 can replace Activin A in non-clonal growth conditions. These defined conditions identify the key molecular pathways required for PGC self-renewal.

## Introduction

Avian species are an important comparative vertebrate model for the study of developmental biology and speciation (Stern, 2005, Zhang et al., 2014). The chicken is also one of the most important agricultural animals, reproducing 59 billion fertile offspring per year (http://faostat3.fao.org/home/E). Primordial germ cells (PGCs) are the precursors to the gametes and central to reproduction. In avian species, the PGCs are formed earlier during embryogenesis than in mammals. Nevertheless, many germ lineage-restricted proteins and pluripotency factors (DDX4, DND, PRDM1, OCT4, NANOG, and SOX2) are common to PGCs in both mammals and birds (Aramaki et al., 2009, Intarapat and Stern, 2013, Lavial et al., 2007, Macdonald et al., 2010, Motono et al., 2008, Tsunekawa et al., 2000). This suggests that, after initial germ cell formation, the genetic mechanisms controlling germ cell self-renewal, growth, and differentiation are similar in these classes of vertebrates (Glover and McGrew, 2012).

In mammalian PGCs, genetic knockout models and short-term PGC culture experiments have implicated the growth factors BMP4, LIF, SCF, retinoic acid, and FGF in early survival and proliferation (Dolci et al., 1991, Dolci et al., 1993, Farini et al., 2005, Matsui et al., 1991). PGCs isolated from mammalian species can only be propagated as lineage-restricted germ cells for short periods in culture (De Felici and McLaren, 1983, Dolci et al., 1991, Durcova-Hills et al., 1998, Farini et al., 2005, Matsui et al., 1991). PGCs from male and female chicken embryos, however, have been propagated long-term in vitro while maintaining lineage specificity and germline competency (van de Lavoir et al., 2006, Song et al., 2014). Chicken PGCs that are isolated from embryonic blood during their migration to the gonad can be expanded extensively in vitro. These germline stem cells form functional gametes and offspring after re-introduction into surrogate host embryos (Choi et al., 2010, Macdonald et al., 2010, Macdonald et al., 2012). Thus, chicken PGCs potentially offer a route to both the cryopreservation, biobanking, of poultry breeds and for the introduction of targeted mutations into the chicken genome (Blesbois et al., 2008, Glover and McGrew, 2012, Park et al., 2014, Petitte, 2006, Schusser et al., 2013).

The development of defined, feeder-free culture conditions will facilitate the in vitro culture of PGCs. The medium for the in vitro propagation of chicken PGCs is ill-defined, containing animal sera, conditioned medium, and a feeder cell layer (van de Lavoir et al., 2006). Here, based on defined serum-free medium conditions for embryonic stem cells (ESCs), we develop defined culture conditions for chicken PGCs and ascertain the minimal signaling pathways necessary for avian germ cell self-renewal. These culture conditions provide insight into the self-renewal of vertebrate PGCs and potential evolutionary changes in this unique population of cells.

## Results

### TGF-β-Signaling Pathways Are Active in Chicken PGCs Both In Vitro and In Vivo

Chicken PGCs isolated from the embryonic blood can be propagated in a complex medium containing fetal bovine serum (FBS), chicken serum, FGF2, and buffalo rat liver (BRL)-conditioned medium on a Sandoz inbred mouse-derived thioguanine-resistant and ouabain-resistant (STO) feeder cell layer (high-serum [HiS] medium) (van de Lavoir et al., 2006). We and others previously have shown that FGF signaling was required for PGC proliferation in vitro (Choi et al., 2010, Macdonald et al., 2010, van de Lavoir et al., 2006). Due to the requirement of FGF2 for PGC growth in vitro, we hypothesized that self-renewal of avian PGCs may be similar to mammalian epiblast stem cells (epiSCs), which require both FGF and TGF-β signaling for self-renewal (Vallier et al., 2005).

**Figure 1:**
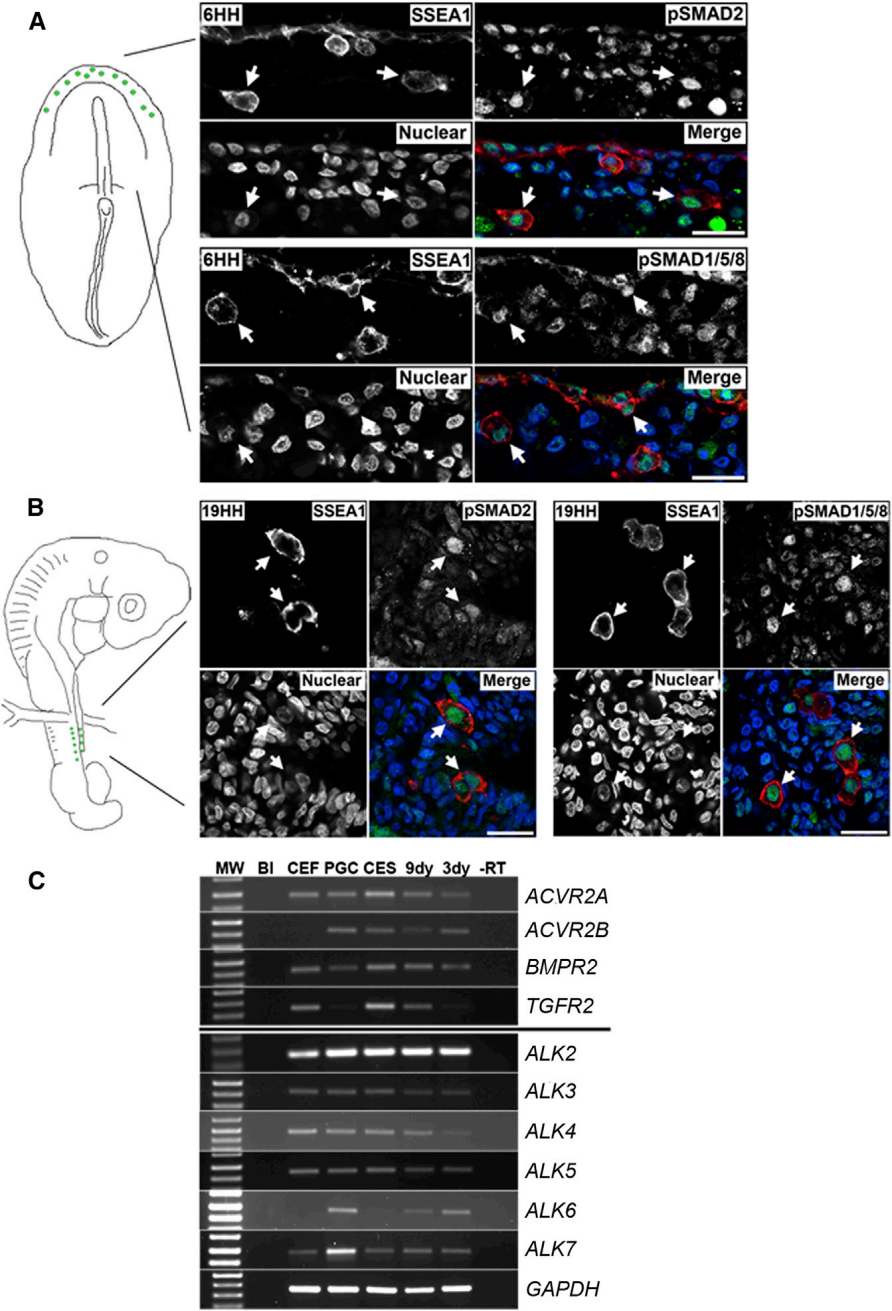
TGF-β-Signaling Pathways Are Active in Chicken PGCs and Needed for PGC Proliferation In Vitro (A and B) Localization of pSMAD2 and pSMAD1/5/8 to SSEA1^+^ migrating PGCs in stage 6 HH chicken embryos and stage 19 HH embryos is shown. Scale bar, 25 μm. (C) RT-PCR was carried out on cDNA from cultured chicken cells and embryonic tissues for TGF-β family receptors. Bl, Blank.

We first investigated whether TGF-β-signaling pathways are active in PGCs in early chicken embryos. Signaling by the Activin/nodal receptors leads to the phosphorylation and nuclear translocation of SMAD2/3 proteins, whereas activation of BMP receptors leads to the phosphorylation and nuclear translocation of SMAD1/5/8 proteins. We assayed pSMAD2 and pSMAD1/5/8 in migratory PGCs at the germinal crescent (stage 6 HH) and in the forming genital ridge (stage 19 HH) (Figures 1A and 1B). Co-immunostaining at these two developmental stages using the germ cell marker SSEA1 revealed the nuclear localization of pSMAD2 and pSMAD1/5/8 in PGCs, indicating that both Activin/nodal- and BMP-signaling pathways are active in migratory PGCs (Figures 1A and 1B). Next we investigated the expression of TGF-β family receptors in chicken PGCs cultured in HiS medium on feeder cells. TGF-β ligands act through heterodimers of TGF-β type I and type II receptors (Shi and Massagué, 2003). An RT-PCR analysis of PGC mRNA revealed that chicken PGCs express the type II receptors *ACVR2A* and *ACVR2B* and the Activin/nodal type I co-receptors *ALK4*, *ALK5*, and *ALK7* (Figure 1C). PGCs also expressed *BMPR2*, the type II BMP receptor, and the type I co-receptors *ALK2*, *ALK3*, and *ALK6*, indicating chicken PGCs could potentially respond to both Activin and BMP ligands.

### Derivation of Male PGCs in Medium Containing FGF, Activin, IGF, and Chicken Serum

**Figure 2:**
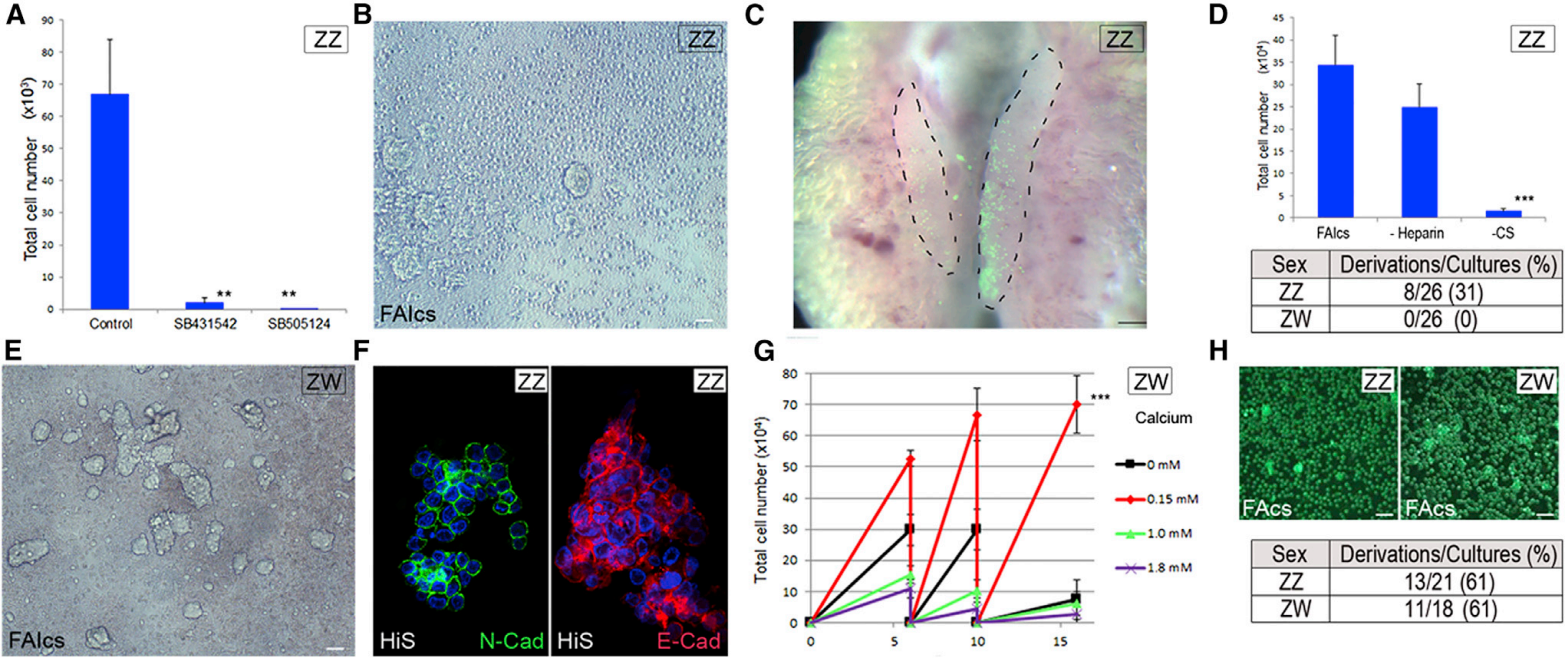
Medium Conditions for Derivation of Both Male and Female PGCs (A) Inhibition of ALK4/5/7 inhibits PGC growth in HiS medium on feeder cells. Approximately 500 PGCs were seeded in growth medium and cultured for 10 days in control condition or in the presence of inhibitor SB0431542 or SB505124. Values represent three independent experiments using three lines of PGCs (SEM; ^∗∗^p < 0.01 versus HiS control). (B) PGC line derivation. Blood from 2.5-day (stage 16 HH) chicken embryos cultured in FAIcs medium for 3 weeks without feeder cells is shown. Scale bar, 75 μm. (C) Male PGCs cultured in FAIcs colonize the gonad in host embryos. Male GFP^+^ PGCs were injected into the vascular system of stage 16 HH embryos, incubated for 3 days, and visualized for GFP fluorescence. Scale bar, 200 μm. (D) (Top) PGC propagation requires the addition of chicken serum. PGCs (5,000) were seeded and cultured for 14 days in FAIcs medium (control) or in medium lacking chicken serum or heparin. Four male PGC lines were assayed in three independent experiments. Error bars, SEM; ^∗∗∗^p < 0.001 with respect to FAIcs. (Bottom) PGC derivation rates are shown. Blood was isolated from single-sexed embryos (stage 16 HH) and cultured for 3 weeks. Cultures containing >50,000 PGCs were scored as positive. (E) Large adherent clusters form in blood cultured from female embryos in FAIcs medium. Scale bar, 75 μm. (F) Male PGC line cultured in HiS media and immunostained for E-cadherin or N-cadherin is shown. Scale bar, 20 μm. (G) PGC number over multiple passages at varying levels of calcium. Female cells (500) were re-plated on days 6 and 10. Data are from three independent experiments. Error bars, SEM; ^∗∗∗^p < 0.001 with respect to other conditions. (H) (Top) Representative example of male and female PGC lines derived from GFP^+^ transgenic embryos is shown. Scale bar, 100 μm. (Bottom) PGC derivation rates in low-calcium medium are shown. Blood was isolated from single-sexed embryos (stage 16 HH) and cultured for 3 weeks. Cultures containing >50,000 PGCs were scored as positive.

Initially we focused our investigation on Activin SMAD2/3 signaling as human ESCs can be cultured in a defined medium containing the growth factors FGF2, Activin A, and either insulin or IGF-1 (Bendall et al., 2007, Eiselleova et al., 2009, Vallier et al., 2005, Wang et al., 2007). Similarly, HiS PGC medium contains conditioned medium from BRL cells, which produce the growth factors Activin and IGF-1 (Kobayashi et al., 2009, Rechler et al., 1979). To demonstrate that the Activin pathway was required for PGC proliferation, we used the chemical inhibitors SB0431542 and SB505124 to inhibit ALK4/5/7 receptors in PGCs in HiS medium on feeder cells (DaCosta Byfield et al., 2004, Inman et al., 2002). We found that inhibition of ALK4/5/7 significantly reduced the in vitro proliferation of PGCs, indicating that the Activin/nodal-signaling pathway is required for PGC self-renewal in serum medium conditions (Figure 2A).

We initially attempted to propagate chicken PGCs without feeder cells or conditioned medium in KO-DMEM basal medium containing the growth factors FGF2, Activin A, and IGF-1 and supplemented with B-27 serum-free supplement, a stem cell supplement containing insulin. The proteoglycan heparin, also was added to increase FGF signaling and reduce cell-cell adhesion (Furue et al., 2008, Tsao et al., 2001). Embryonic blood (1 μl) from a single chicken embryo was placed in a single well and cultured for 3 weeks, and the PGCs present at the end of this culture period were counted. In these conditions, male (ZZ) PGCs initially could be propagated but subsequently could not be expanded in culture (data not shown). However, upon the addition of 0.2% chicken serum to this medium (FAIcs), male PGCs could be propagated and expanded indefinitely in culture (Figures 2B and 2D), and also they could be migrated to the gonad when injected into host embryos (Figure 2C). However, only PGC lines isolated from male embryos could be derived in this culture medium (Figure 2D).

### In Vitro Culture of Female PGCs in Low-Serum Medium Requires Specific Physiochemical Conditions

In derivations started from female embryonic blood, cells that morphologically resembled PGCs forming large adherent clusters were apparent after 1 week in culture (Figure 2E). A similar clustering of female PGCs in HiS medium also has been reported (Song et al., 2014). To delineate medium conditions permissive for the self-renewal of female PGCs, we first assayed the osmolality of embryonic blood at the developmental stage of PGC migration through the circulatory system (stage 16HH). We found that chicken embryonic blood has a lower osmolality (260 mOsm/kg) compared to most basal media (Figure S1A). Male PGCs were cultured in FAIcs at varying osmolalities without feeder cells and assayed for cell proliferation (Figure S1B). We determined that a medium of osmolality less than 300 mOsm/kg was required for PGC growth, and PGC proliferation was optimal at an osmolality of 250 mOsm/kg.

Previous research has demonstrated that mouse PGCs express the homodimeric calcium-dependent cell adhesion molecules E- and N-cadherin (Bendel-Stenzel et al., 2000, Di Carlo and De Felici, 2000, Okamura et al., 2003). We assayed male PGC lines cultured in HiS medium, as these cells showed a greater propensity to grow in clusters, for the expression of these molecules, and we observed expression of E-cadherin and N-cadherin on the cell surface (Figure 2F). We hypothesized that lowering calcium levels could prevent cell-cell interactions and the apparent clustering of female PGCs without affecting cell viability (Peshwa et al., 1993). We found that female (ZW) PGCs could be propagated as dispersed single cells in a basal medium of 250 mOsm/kg containing physiologically lower levels of calcium (0.15 mM) (Figure 2G). Using this modified basal medium (avian KO-DMEM) containing FGF2, Activin A, IGF-1, and chicken serum (FAIcs medium), both male and female PGC cell lines could be derived from the blood of single embryos and propagated and expanded indefinitely in suspension without feeder cells (Figure 2H). Avian KO-DMEM was used as the basal medium for all subsequent experiments.

### Activin-, FGF-, Insulin-, and BMP-Signaling Pathways Are Active in PGCs

**Figure 3:**
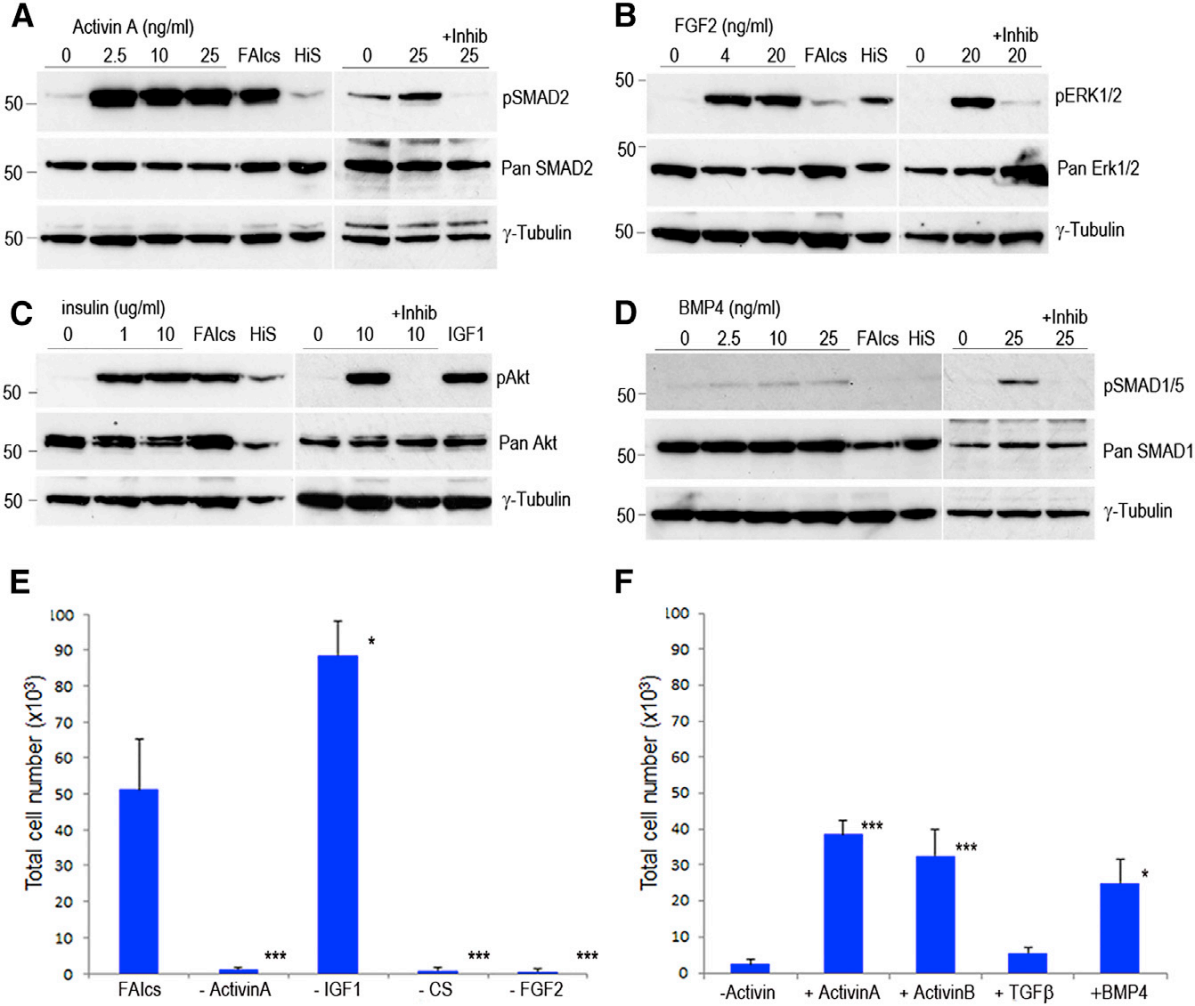
FGF, Insulin, and Either Activin or BMP4 Are Essential for PGC Self-Renewal (A–D) PGCs were starved for 24 hr, pre-incubated with the indicated inhibitor for 30 min, and then induced with the given concentrations of growth factors for 15 min. Cells were lysed and proteins were analyzed by western blot analysis. Blots represent one of two independent experiments using a male or a female PGC line. FAIcs, control cells in FAIcs medium; HiS, control cells in full-serum medium; Inhib, Inhibitor. (A) Activin A induction of pSMAD2 in PGCs is inhibited by SB0431542. (B) FGF2 induction of pERK1/2 in PGCs is inhibited by PD173074. (C) Insulin induction of pAkt in PGCs is inhibited by BMS 536924. (D) BMP4 induction of pSMAD1/5 in PGCs is inhibited by LDN-193189. (E and F) PGC number over 10 days in culture (500 were plated on day one). Each cell treatment was assayed on three technical replicates on three different PGC lines (one male and two female). (E) Control medium containing FAIcs compared with FAIcs after removal of one component (−) is shown. Error bars, SEM; ^∗∗∗^p < 0.001 with respect to FAIcs samples. (F) Control medium containing FGF2, IGF-1, and chicken serum (FIcs) compared with FIcs medium after the addition of 25 ng/ml Activin A, Activin B, or TGF-β1 is shown. Error bars, SEM; ^∗^p < 0.05 and ^∗∗∗^p < 0.001 with respect to −Activin samples.

To confirm that the FGF-, Activin-, and insulin-signaling pathways were active in chicken PGCs, we carried out growth factor induction assays. PGCs were cultured overnight in a medium lacking all serum and growth factors. PGCs were then induced with the respective growth factors for 15 min, with or without the addition of a chemical inhibitor of the corresponding receptor, and analyzed by immunoblotting. As stated above, Activin acts through heterodimers of type I and type II serine/threonine kinase receptors to phosphorylate SMAD2/3. The addition of Activin A led to increased phosphorylation of SMAD2 (Figure 3A). This phosphorylation was ablated by the addition of the type II receptor inhibitor SB0431542. FGF signals through the FGF receptors to phosphorylate the serine/threonine kinase ERK1/2. Chicken PGCs expressed the FGF receptors 1, 2c, and 4 (Figure S2A). We found that the addition of FGF2 ligand led to an increase in phosphorylation of ERK1/2 (Figure 3B). This phosphorylation was ablated by the presence of the FGF receptor inhibitor PD173074. Insulin is a pleiotropic growth factor acting through the insulin receptor (INS-r) and IGF1 and 2 receptors. Chicken PGCs expressed *INSR*, *IGF1R*, and *IGF2R* receptors (Figure S2A). Insulin acts on many intracellular signaling pathways (Taniguchi et al., 2006) and a central downstream target of insulin signaling is the serine/threonine kinase Akt, involved in cell proliferation and survival. The addition of insulin or IGF-1 led to an immediate phosphorylation of Akt in chicken PGCs (Figure 3C). The addition of the pan-insulin/IGF receptor inhibitor BMS 536924 ablated the phosphorylation of Akt. These data indicate that chicken PGCs respond to the growth factors FGF2, Activin, and insulin and phosphorylate downstream signaling targets.

Our in vivo analysis indicated that the SMAD1/5/8 pathway also was active in migrating PGCs (Figures 1A and 1B). In the mouse, the growth factor BMP4 is required for the initial formation of the germ cell lineage and PGC survival (Farini et al., 2005, Lawson et al., 1999, Ying et al., 2001). To demonstrate that chicken PGCs can respond to BMP4, PGCs were induced with BMP4 and assayed for phosphorylation of SMAD1/5, the principal effector of BMP signaling (Figure 3D). The addition of BMP4 led to an increase in phosphorylation of SMAD1/5, which was ablated by the addition of the BMP4 receptor inhibitor LDN-193189. We assayed whether Activin and BMP4 activated the reciprocal SMAD-signaling pathways, as has been reported in some cell lineages (Daly et al., 2008, Upton et al., 2009). We were unable to detect cross-activation between the Activin and BMP ligands and the other signaling pathways investigated here (Figure S3). Induction with FGF2 ligand led to a slight but reproducible phosphorylation of Akt, indicating FGF receptor signaling also leads to activation of Akt in PGCs (Figure S3). These results suggest that Activin A and BMP4 signal through independent SMAD regulatory molecules in chicken PGCs.

Next we systematically assayed the individual growth factors required for PGC proliferation in FAIcs medium. Single growth factors were removed from the medium and cell proliferation was assayed after 10 days in culture. These experiments confirmed that FGF2 and Activin A were required for PGC proliferation, as was the requirement for chicken serum (Figure 3E). As the B-27 supplement contains the peptide hormone insulin, we assayed if addition of IGF-1 was required for PGC proliferation (Figure 3E). We found that additional IGF-1 was not required for PGC proliferation and this growth factor was removed in subsequent experiments (FAcs medium). We next asked if insulin was required for PGC proliferation by removing insulin from the B-27 supplement (Figure S2B). We found that PGCs did not proliferate without the addition of insulin to the medium. The growth factors Activin A and Activin B were equally effective for PGC proliferation, but TGF-β1 could not replace Activin in the culture medium (Figure 3F). As the BMP-SMAD1/5/8 pathway was active in PGCs both in vitro and in vivo, we asked if BMP4 could replace Activin A in the culture medium. Surprisingly, we found that BMP4 was sufficient for PGC proliferation in vitro (Figure 3F).

### Male and Female PGCs Cultured in Activin A, FGF2, and Insulin Are Germline Competent

**Figure 4:**
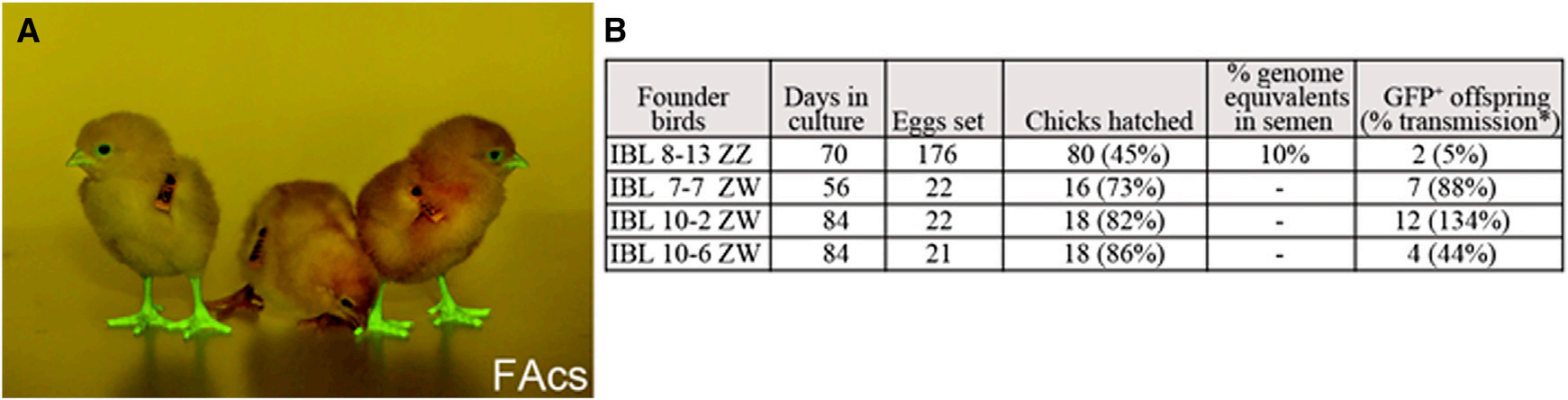
Chicken PGCs Propagated in Activin Are Germline Competent (A) PGCs cultured in FAcs are germline competent. One male and one female GFP^+^ PGC line were mixed, injected into host embryos, hatched, and raised to sexual maturity. The photograph shows several offspring of a surrogate host hen imaged for GFP fluorescence. (B) Frequency of germline transmission from donor GFP^+^ male and female PGCs in male and female surrogate host chickens is shown. ^∗^The actual transmission rate is double the observed number of GFP^+^ chicks due to meiotic reduction of the heterozygous GFP transgene.

To demonstrate that chicken PGCs propagated in FAcs medium were germline competent, male and female GFP^+^ PGC lines were derived from single embryos in FAcs medium and cryopreserved after 6 weeks in culture. The PGC lines were then thawed and re-cultured. Male and female PGC lines were mixed in equal numbers and injected into host embryos. The host embryos were hatched, raised to sexual maturity, and mated to wild-type chickens. It has been demonstrated previously that germline transmission of PGCs is only possible in host birds of the same sex as the injected germ cells (Macdonald et al., 2010, van de Lavoir et al., 2006). GFP^+^ offspring were obtained from both male and female surrogate host chickens (Figures 4A and 4B), indicating that both male and female PGCs propagated in FAcs medium were germline competent.

### Ovotransferrin Replaces All Serum Requirements

**Figure 5:**
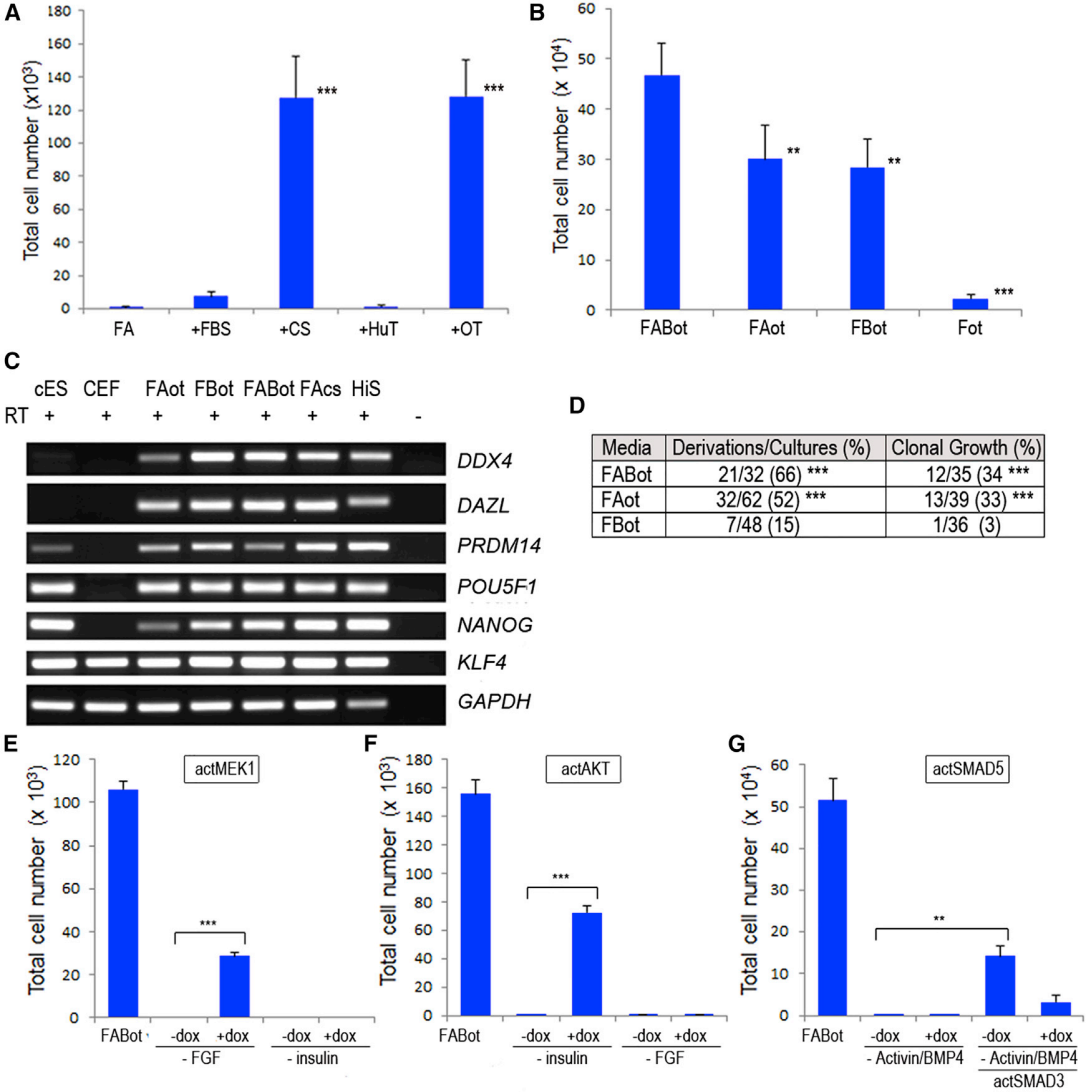
PGCs in Defined Medium Can Be Cultured Clonally in Activin, but Not BMP4 (A) Ovotransferrin (OT) replaces chicken serum in culture medium. PGCs (1,000) were cultured in FA medium with the addition of 0.2% fetal bovine serum (FBS), 0.2% chicken serum (CS), and 10 μg/ml human transferrin (HuT) or 10 μg/ml OT for 10 days. Error bars, SEM; ^∗^p < 0.05 and ^∗∗∗^p < 0.001 with respect to FA samples. Each cell treatment was assayed in three independent experiments on a male and a female PGC line. (B) PGCs can be propagated in Activin or BMP4 in defined medium conditions. PGC number over 14 days in culture (500 were plated on day 1) is shown. Each cell treatment was assayed on six different PGC lines (four male and two female) in three independent experiments. Error bars, SEM; ^∗∗^p < 0.01 and ^∗∗∗^p < 0.001 with respect to FABot sample. (C) PGCs express pluripotency and germ cell markers in Activin- or BMP4-defined culture medium. RT-PCR analysis of cDNA prepared from a PGC line cultured in FABot, FAot, or FBot is shown. FAcs and HiS are shown as positive controls. Image represents one of two independent experiments using two male PGC lines. CEF, chick embryonic fibrobast; cES, chick embryonic stem cell. (D) Activin is sufficient for derivation and clonal growth of PGCs. Blood was isolated from single embryos (stage 16 HH) and cultured for 3 weeks. PGCs were counted and cultures containing >50,000 cells were scored as positive. Cells from a male or a female PGC line derived in FABot were diluted to one cell/2 μl in Fot, and single cells were plated and cultured for 3 weeks in FABot, FAot, or FBot. PGCs were counted and cultures containing >50,000 cells were scored as positive. ^∗∗∗^p < 0.001 with respect to FBot samples using two-tailed Fisher’s exact test. (E–G) FGF2, insulin, and Activin can be replaced by the corresponding downstream effectors. PGCs (500), cultured in FABot, were seeded in a well and the indicated growth factors were removed. Doxycycline (dox) was added and cells were propagated for 10 days (A and B) or 12 days (C) and then counted. (E) PGCs containing a tet-inducible constitutively active MEK1 construct proliferate in the absence of FGF2. (F) PGCs containing a tet-inducible constitutively active AKT construct proliferate in the absence of insulin. (G) PGCs containing constitutively active SMAD3 and tet-inducible constitutively active SMAD5 constructs proliferate in the absence of Activin and BMP4. Each cell treatment was assayed using two different PGC lines (one male and two female) in three independent experiments. ^∗∗∗^p < 0.001 and ^∗∗^p < 0.01 with respect to the indicated samples.

FGF2, Activin, and insulin growth factors are sufficient for PGC self-renewal, but only in the presence of a low concentration (0.2%) of chicken serum (Figures 2D and 3E). This serum requirement could not be replaced by the addition of FBS (Figure 5A). Chicken serum contains numerous growth factors, avian-specific cytokines, and serum components. A principal component of animal sera is transferrin, an iron-binding glycoprotein present at micromolar concentrations in animal sera and a key component in many serum-free supplements, such as B-27 (Brewer et al., 1993, Ponka, 1999). A species specificity for the cellular uptake of Fe^2+^-transferrin has been reported between avian and mammalian transferrins (Shimo-Oka et al., 1986, Sorokin and Morgan, 1988). To address whether avian transferrin could replace chicken serum, PGCs were cultured in a medium lacking chicken serum and containing chicken transferrin (ovotransferrin [OT]) or human transferrin. We found that the addition of OT, but not human transferrin, could replace chicken serum in FAcs medium (Figure 5A). Thus, chicken PGCs can be propagated in defined medium conditions containing FGF2, Activin, insulin, and OT (FAot), further indicating these growth factors alone are sufficient for PGC self-renewal.

### Activin Is Sufficient for Clonal Growth of PGCs in Defined Medium Conditions

Using defined medium conditions, we re-investigated the relationship between Activin and BMP in chicken PGC proliferation. We first asked whether there was a quantitative difference in cell proliferation in the presence of both Activin A and BMP4. We found no significant differences in cell proliferation between PGCs cultured in either Activin A or BMP4, but cells did have increased proliferation with both growth factors present (Figure 5B). PGCs cultured under each of these three media conditions expressed pluripotency factors associated with both ESCs and the germ cell lineage (Figure 5C).

To delineate if Activin A and BMP4 were both sufficient for the derivation of PGC lines, embryonic blood from single chicken embryos was placed in single wells and the resulting PGCs were counted after 3 weeks. We found that both male and female PGC lines could be derived in these three medium conditions. PGC lines could be derived in medium containing BMP4 alone, but the derivation rate was significantly lower than the derivation rate in medium containing Activin A (Figure 5D).

We finally asked whether Activin A and BMP4 could both support PGC propagation in clonal growth conditions. A single PGC was plated into a single well and cultured for 21 days. Under clonal growth conditions, PGCs could be propagated in medium supplemented with Activin A, but not in medium supplemented with BMP4 alone (Figure 5D). No difference in clonal growth was observed between medium containing Activin A alone and medium containing both Activin A and BMP4. These results indicate that FGF2, insulin, and Activin A can sustain the self-renewal and clonal growth of chicken PGCs under permissive physiochemical medium conditions and BMP4 can replace Activin A under non-clonal growth conditions.

### Constitutively Active AKT, MEK1, and SMAD3 Can Replace the Cognate Growth Factors

We lastly studied if FGF2, insulin, and Activin A/BMP4 could be replaced by activating the downstream signaling molecules of the cognate-signaling pathways. Transposon vectors containing an AKT or MEK1 tetracycline-inducible transgene were stably transfected into PGCs and selected (Glover et al., 2013). PGCs were plated into a well in FABot medium, the candidate growth factor was removed, and doxycycline (dox) was added to induce expression of the specific signaling protein under investigation. Cells were cultured for 10 days and proliferation was assayed. PGCs containing a constitutively active AKT protein proliferated after the removal of insulin from the culture medium (Figure 5E). Although we detected phosphorylation of Akt after the induction with FGF2 (Figure S3), PGCs containing the constitutively active AKT protein did not proliferate after the removal of FGF2. PGCs containing a constitutively active MEK1 construct proliferated after the removal of FGF2, but did not proliferate in the absence of insulin in the culture medium (Figure 5F).

Finally, transposons containing a constitutively expressed, constitutively active SMAD3 protein and an inducible constitutively active SMAD5 construct were both stably introduced into PGCs. Activin A and BMP4 ligands were removed and cells were cultured with and without dox for 12 days. PGCs containing a construct expressing a constitutively active SMAD3 proliferated in culture in the absence of Activin A and BMP4 (Figure 5G). Constitutively active SMAD5 on its own or combined with constitutively active SMAD3, however, did not rescue PGC propagation in culture. These results confirm that avian germ cell self-renewal can be met by the growth factors insulin, FGF2, and Activin by signaling through their cognate receptors and activating ERK1/2, Akt, and SMAD3 intracellular signaling molecules.

## Discussion

**Figure 6:**
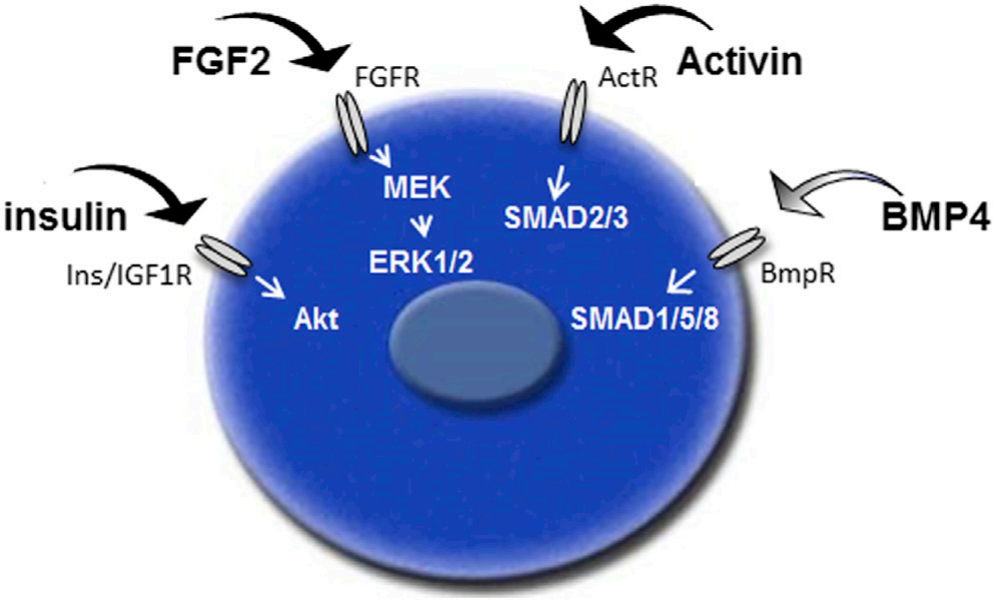
Model for Avian Primordial Germ Cell Self-Renewal FGF2, insulin, and Activin growth factors signaling through their cognate receptors are sufficient for chicken PGC self-renewal. BMP4 can replace Activin A in non-clonal growth conditions.

Defined culture conditions are instructive to delineate the minimal extrinsic signals needed for stem cell renewal (Ying et al., 2003). In serum-free, feeder cell-free, and physiochemically permissive medium conditions, we found that FGF2, insulin, and Activin ligands were sufficient for the derivation, expansion, and clonal growth of chicken PGCs. These ligands, signaling through their cognate receptors and downstream signaling pathways, define the signals needed for the self-renewal of a chicken PGC (Figure 6). TGF-β signaling through SMAD2/3 molecules, rather than SMAD1/5/8, appears to be more crucial to PGC self-renewal, as a constitutively active SMAD3 protein was able to rescue PGC growth in the absence of both BMP4 and Activin (Figure 5G). BMP4 can sustain self-renewal and expansion of PGC cultures, but not under clonal conditions, which suggests signaling through cell-cell interactions, reduced through lowering calcium levels, may be important for chicken PGC proliferation and survival. It is also possible that Activin A induces expression of a key downstream effector molecule that permits survival under clonal growth conditions. Nevertheless, a culture medium containing both Activin and BMP4 ligands may more generally reflect the in vivo environment, as both signaling pathways were active in migratory chicken PGCs in ovo, as evidenced by the phospho-SMAD1/5/8 and phospho-SMAD2 staining observed in the chicken embryo (Figures 1A and 1B).

PGCs in birds are first found as a cluster of cells in the center of the blastoderm (Eyal-Giladi et al., 1981, Tsunekawa et al., 2000). From here, PGCs migrate to the germinal crescent anterior of the neural plate, enter the forming vascular system, are transported to the posterior lateral plate mesoderm, and finally migrate to the forming genital ridge (Nakamura et al., 2007, Nieuwkoop and Sutasurya, 1979). The complex migration path of avian PGCs may necessitate the ability to respond to multiple TGF-β-signaling ligands that vary spatially across the developing embryo.

A striking finding from this work is that, in serum-free conditions, chicken PGCs can survive and proliferate in the absence of stem cell factor (SCF). SCF (c-KIT ligand) is a requisite survival factor for mouse PGCs at early embryonic stages and for survival in short-term culture experiments (Gu et al., 2009, Huang et al., 1990, Matsui et al., 1992). SCF signaling through the c-KIT receptor was shown to phosphorylate AKT and inhibit germ cell apoptosis (Blume-Jensen et al., 2000, Pesce et al., 1993). It is possible that insulin may replace SCF in our culture conditions.

The Activin/TGF-β- and BMP-signaling pathways generally antagonize each other (Goumans et al., 2003), but in certain cell types both signaling pathways can be activated by a common ligand (Daly et al., 2008, Upton et al., 2009). In chicken PGCs, both pathways are active and the presence of both Activin and BMP4 provides the optimum conditions for PGC derivation and growth. This suggests that some interactions occur between these pathways most likely at a molecular level, although these interactions could not be detected in our experiments, which focused on the phosphorylation of signaling proteins.

A potential downstream target of Activin signaling is the pluripotency factor NANOG. Activin/TGF-β signaling is known to regulate NANOG expression in human ESCs and mouse epiSCs (Vallier et al., 2009, Xu et al., 2008), and NANOG is also essential for germ cell specification and survival in mice (Chambers et al., 2007, Yamaguchi et al., 2009). In the chicken, *NANOG* is expressed in PGCs and also in the early epiblast (Cañón et al., 2006, Lavial et al., 2007, Shin et al., 2011). NANOG is required to maintain pluripotency in chicken ESCs (Lavial et al., 2007), and its expression in the epiblast is regulated by Activin/TGF-β signaling (Shin et al., 2011). BMP signaling is essential for PGC specification in mice (Lawson et al., 1999, Ying et al., 2001), urodele amphibians (Chatfield et al., 2014), and crickets (Donoughe et al., 2014), suggesting an evolutionarily conserved role for BMPs in inductive PGC specification. However, our findings demonstrate that chicken PGCs, which evidence suggests are specified through the inheritance of maternal determinants (Tsunekawa et al., 2000), also use BMP/SMAD1/5/8 signaling for self-renewal. This indicates that BMP4/SMAD1/5/8 signaling is not solely restricted to animals in which PGCs are specified through epigenesis. The chicken is an important comparative animal model for development biology and is also a major source of farmed animal meat and egg production for human consumption (Herrero et al., 2013, Stern, 2005). The defined medium conditions shown here will aid the development of PGC biobanks and efforts in gene editing of the chicken genome. Finally, further investigation into the core signaling networks that underpin chicken germ cell survival and proliferation will provide a greater understanding of the biology of vertebrate germ cells.

## Experimental Procedures

### Cell Culture Medium

Media components were purchased from Life Technologies unless specifically cited. PGC HiS serum culture medium contained 7.5% FBS (ESC tested, PAA Laboratories), 2.5% chicken serum (Biosera), 2.0 mM GlutaMax 1× NEAA, 0.1 mM β-mercaptoethanol, 1× nucleosides, 1× penicillin-streptomycin and 2 ng/ml human recombinant FGF2 (R&D Biosystems), and 30% conditioned medium (KO-DMEM conditioned on BRL cells for 4 days) in knockout-DMEM. PGCs grown in HiS medium were cultured on irradiated STO feeder cells (3.0 × 10^4^ cells per cm^2^).

Avian KO-DMEM basal medium is a custom modification of knockout-DMEM (250 mOsm/kg, 12.0 mM glucose, and calcium chloride free) produced by Life Technologies. FAcs medium and derivatives of this medium contained avian KO-DMEM basal medium, 1× B-27 supplement, 2.0 mM GlutaMax, 1× NEAA, 0.1 mM β-mercaptoethanol, 1× nucleosides, 1.2 mM pyruvate, 0.2% ovalbumin (Sigma), and 0.2% sodium heparin (Sigma). Human Activins A and B, 25 ng/ml (PeproTech); human BMP4, 25 ng/ml (PeproTech); human TGF-β1, 25 ng/ml (PeproTech); human FGF2, 4 ng/ml (R&D Biosystems, Sigma); and human IGF-1, 25 ng/ml (R&D Biosystems) stocks were prepared following each manufacturer’s protocols and used at the indicated final concentrations. OT (Sigma) was used at 10 μg/ml. Osmolality of freshly isolated embryonic blood plasma samples (20 μl) was determined using an Advanced 3MO micro-osmometer (Advanced Instruments).

PGC lines were derived by placing 1 μl blood isolated from stage 15–16 (H&H) embryos (ISA Brown layer line) in 300 μl medium in a 48-well plate without feeder cells. The sex of the donor embryo was determined as described previously (Macdonald et al., 2010). One-third of the medium was changed every 2 days. When total cell number reached 1.0 × 10^5^, the total volume of medium was changed every 2 days and cells were propagated at 2–4.0 × 10^5^ cells/ml medium in a 24-well plate. Cells were frozen in avian KO-DMEM containing 5% DMSO/4% chicken serum or cryoMAXX solution (PAA Laboratories) and stored at −150°C.

The p values for cell culture experiments were determined using the general linear model for ANOVA, and multiple comparisons were conducted using Tukey’s post hoc test unless otherwise stated.

### Western Blot

For induction and inhibition assays, PGCs were starved overnight in serum-free basal media containing 1× insulin-free B-27 supplement. Following overnight starvation, PGCs were either directly induced with growth factor for 15 min or pre-incubated with inhibitors for 30 min before growth factor addition and assayed as described in the Supplemental Experimental Procedures.

### Embryo Injection of Cultured PGCs and Germline Transmission

For germline transmission, one male and one female cell line from CAG-GFP embryos were expanded in culture in FAcs medium for 27 days and cryopreserved. Cells were thawed after storage for 37 days at −150°C. PGCs were cultured for 4–8 weeks and counted. Then, 5,000–6,000 cells (1:1 mixture of male and female cells) were injected into stage 16 HH host embryos and incubated until hatching using the surrogate shell culture system (Macdonald et al., 2010). Three injection experiments were carried out and ten of 31 injected embryos survived to hatching. The hatched chicks were raised to sexual maturity, and genomic DNA extracted from the semen of adult roosters was screened by semiquantitative PCR to identify GFP transgenic DNA in the semen (McGrew et al., 2004). One rooster was crossed to wild-type hens and the offspring were screened for GFP fluorescence to identify germ cell-derived offspring. Three hens were mated to wild-type roosters and the offspring were screened for GFP fluorescence to identify germ cell-derived offspring. Animal experiments were conducted under UK Home Office license.

## Author Contributions

J.W., M.W., J.D.G., J.B., L.T., A.S., and M.J.M. conducted experiments. J.W., M.W., J.D.G., and M.J.M. prepared the figures. J.W., M.W., J.D.G., J.B., L.T., A.S., and M.J.M. analyzed the data. J.W., M.W., J.D.G., J.B., L.T., A.S., P.K., and M.J.M. planned the project and wrote the manuscript. A.S., P.K., and M.J.M. supervised the project.

## Supplemental Information

Document S1. Supplemental Experimental Procedures and Figures S1–S3

Document S2. Article plus Supplemental Information
