# Supplementary material for: FGF, Insulin, and SMAD Signaling Cooperate for Avian Primordial Germ Cell Self-Renewal
Source: Stem Cell Reports. 2015 Nov 19;5(6):1171–82. doi: 10.1016/j.stemcr.2015.10.008 (PMC4682126; doi:10.1016/j.stemcr.2015.10.008)
Supplement: Document S1. Supplemental Experimental Procedures and Figures S1–S3 [file mmc1.pdf]

Stem Cell Reports, Volume *5*

Supplemental Information

FGF, Insulin, and SMAD Signaling Cooperate for Avian Primordial Germ Cell Self-Renewal

Jemima Whyte, James D. Glover, Mark Woodcock, Joanna Brzeszczynska, Lorna Taylor, Adrian Sherman, Pete Kaiser, and Michael J. McGrew

Supplemental Figures

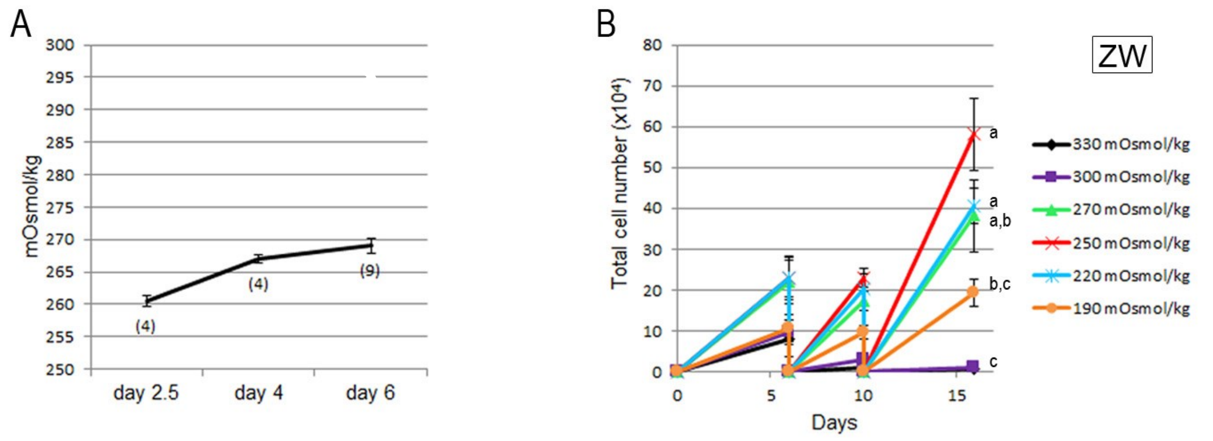

Figure S1. Related to Figure 2. Low osmolality conditions are permissive for chicken PGC proliferation

(A) Osmolality of chicken embryonic blood. Blood was isolated from the indicated developmental stages and the osmolality was assayed. Error bars, S.E.M.

(B) PGC number over multiple passages at varying medium osmolality. 500 male cells were re-seeded on each passage on days 6 and 10. Data from three independent experiments; Error bars, S.E.M., conditions not sharing a letter are significantly different ($p < .01$).

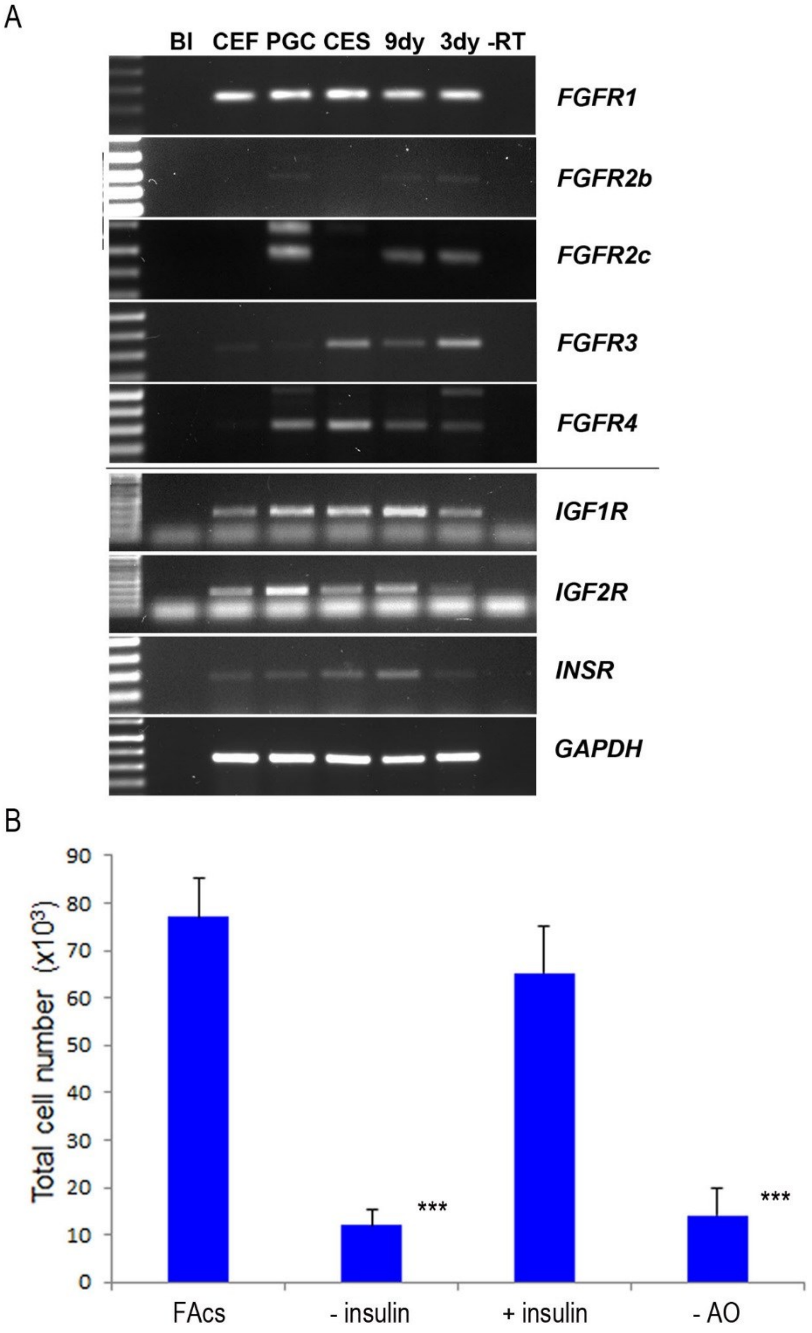

Figure S2. Related to Figure 3. Insulin and FGF signalling in chicken PGCs

(A) Chicken PGCs express receptors for the insulin and FGF signalling pathways. RT-PCR was carried out on cDNA from cultured chicken cells lines and embryonic tissues. Bl, Blank CEF, chick embryonic fibrobasts; CES, chick embryonic stem cells. Control GAPDH panel is the same as in Figure 1C.

(B) Insulin is necessary for PGC survival and proliferation. PGCs (1000) were seeded into a well, cultured for 10 day and counted. Control, FAcs medium compared with FAcs medium after removal of insulin.

Each cell treatment was assayed on three different PGC lines (1 male and two female) in three independent experiments. Error bars, S.E.M. **, $p<0.01$; ***, $p<0.001$ with respect to control.

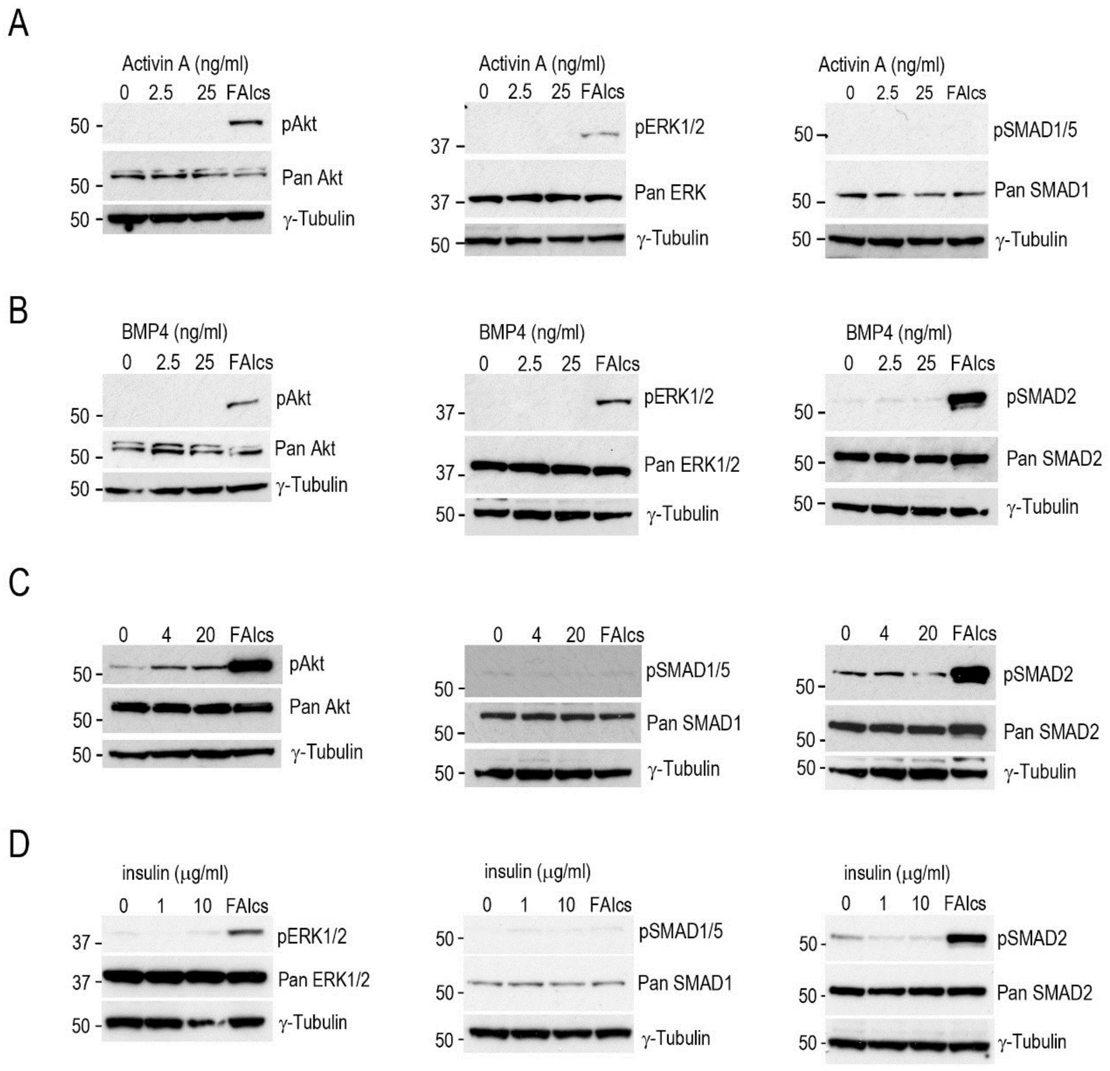

Figure S3. Related to Figure 3. Activin and BMP ligands display pathway specificity.

PGCs were starved for 24 hours and then induced with the given concentrations of growth factors for 15 minutes. Cells were lysed and proteins were analysed by Western blot analysis. Blots represent one of two independent experiments using a male or a female PGC line. FAIcs; control cells in FAIcs medium.

(A) PGCs were induced with Activin A and assayed for and pAkt, pERK1/2, and pSMAD1/5.

(B) PGCs were induced with BMP4 and assayed for pAkt, pERK1/2, and pSMAD2.

(C) PGCs were induced with FGF2 and assayed for pAkt, pSMAD1/5, and pERK1/2

(D) PGCs were induced with insulin and assayed for pERK1/2, pSMAD1/5, and pSMAD2.

Supplemental Experimental Procedures

Inhibitors and antibodies

SB0431542 and SB505124 (Sigma), LDN-193189 and PD173074 (StemGent) and BMS 536924 (Tocris Bioscience) were dissolved at 10 mM in DMSO and diluted at the given concentrations in cell culture medium. For inhibition assays SB0431542 was added at 10 µM final concentration (f.c.), LDN-193189 was added at 100 nM f.c., PD173074 was used at 100 ng/ml f.c. and BMS 536924 was used at 10 µM f.c.

Primary antibodies and dilutions used for immunoblot analysis were rabbit anti-human Phospho-SMAD1/5 (Ser 463/465) (1:1000; #9516), rabbit anti-human phospho SMAD2 (Ser 465/467) (1:1000; #3108), rabbit anti-human SMAD1 (1:1000; #6944), rabbit anti-human Smad2 (1:1000; #5339), rabbit anti-human phospho Akt (Ser 473) (1:2000; #4060), mouse anti-human pan Akt (1:2000; #2920), rabbit anti-human phospho p44/42 MAPK (ERK1/2) (Thr202/Tyr204) (1:2000; #4370), rabbit anti-human P44/42 MAPK (ERK1/2) (1:1000; #4695) from Cell Signalling Technologies and goat anti-human gamma tubulin (1:1000; sc-7396) from Santa Cruz Biotechnology Inc. Primary antibodies used for immunostaining were rabbit anti-human phospho SMAD2 (Ser 465/467; #3108) and rabbit anti-human phospho SMAD1/5/8; #9511, (Cell signalling Technologies), mouse anti-chicken N-cadherin (6B3; Developmental Studies Hybridoma Bank) and rabbit anti-mouse E-cadherin (610181; BD Biosciences).

Western blot

Following inductions, PGCs were washed, extracted in 25µl lysis buffer on ice and quantitated using the DC protein assay (BioRad). 10µg of protein lysate was electrophoresed under reducing conditions on 12% Bis-Tris gels and transferred to a nitrocellulose membrane. Membranes were blocked in 5% milk and then incubated with the primary antibody overnight at 4 °C (1:2000). Membranes were incubated with horseradish peroxidise (HRP)-conjugated secondary antibodies (DAKO, UK) (1:2000) for 2 hr and then developed with Novex ECL chemiluminescent substrate reagent kit (Life Technologies) and visualised using Hyperfilm ECL (Amersham).

Immunostaining

PGCs (150,000) or cryo-embedded embryo sections were fixed in 4% paraformaldehyde, permeabilised with 0.1% Triton X-100 in PBS, and washed 3x with PBS containing 0.1% Tween-20 (PBT). Samples were incubated with the primary antibody dissolved in 5% sheep serum overnight at 4°C. Samples were washed with PBT for 1 hr, and incubated with Alexa-Fluor 488 conjugated or Alexa-Fluor 555 conjugated secondary antibodies (Life Technologies) (1:500 dilution) for 1 h at room temperature. Samples were washed PBT for 1 hr, and stained with Hoechst (Sigma) to visualize nuclei.

Cells were mounted under coverslips in PBS and cryosections were mounted in hydromount (National Diagnostics) and visualized using a Zeiss LSM 710 inverted confocal microscope. Images were captured using Zen Black software (Zeiss).

Expression vector cloning

A cDNA fragment encoding a constitutively active Smad3 was cloned into the *Eco*R1 site of pbCAG-IRES-Neo to generate pbCAG-Smad3. A constitutively active Smad5 (Le Dreau et al., 2012) and constitutively active Mek1 (Mansour et al., 1994) were cloned into the *Hpa*1 site of pbTet-On empty vector (Glover et al., 2013) to generate pbTet-ON SMAD5 and pbTet-ON MEK1. The pb Tet-ON Akt was from (Glover et al., 2013). 1 µg of the transposon vector and 1 µg of CAG piggybac hypertransposase were transfected into PGCs and selected as previously described (Macdonald et al., 2012).

RT PCR analysis

Total RNA was isolated from cultured PGCs using the RNeasy Mini Kit (Qiagen). Total RNA (500 ng) was reverse-transcribed and subjected to polymerase chain reaction (PCR). PCR conditions were 94 °C for 5 min, 94 °C for 30 s, 60 °C for 30 s, and 72 °C for 30 s for 30 cycles. PCR conditions for Klf4 were 95 °C for 4 min, 95°C for 30 s, 50 °C for 30 s, and 72 °C for 1 min for 34 cycles Reaction products were resolved using a 2.5% ultrapure agarose (Invitrogen) gel electrophoresis run at 100 V for 1 h in 1X TAE (Tris base, acetic acid, EDTA) buffer, and visualised using a transilluminator. Primer sequences are listed in supplemental experimental procedures.

Primer sets used for PCR

GAPDH: fwd, CCTCTCTGGCAAAGTCCAAG rev, CATCTGCCCATTTGATGTTG;
KLF4: fwd, AGCTCTCATCTCAAGGCACA rev, GGAAAGATCCACTGCTTCCA;
DAZL: fwd, TCCCAGAGCCCACACAGATG rev, AAGTGATGCGCCCTCCTCTC;
NANOG: fwd, AGCAGACCTCTCCTTGACCA rev, TTCCTTGTCCCACTCTCACC;
POU5F1: fwd, GGCTCAATGAGGCAGAGAAC rev, GGACTGGGCTTCACACATTT;
PRDM14: fwd, TGTTCGCCTACCGCTACTACCG rev, AGTGCTGGCGGAGTGTGTGTG;
SOX2: fwd, GTGAACCAGAGGATGGACAGTTACG rev, TGCGAGCTGGTCATGGAGTTG;
DDX4: fwd, TCCATCTTTGCATGTTATCAGTCAGG rev, AATCCCGCCCTGCTTGTATAACAG;
FGFR1: fwd, GTCTCAGACGCACTCCCTTC rev, GTCAGGCTTGAACTCCTTGC
FGFR2B: fwd, TTACCTTCAGGTTTTAAAGCATTC rev, TGGCAGTTCATATTCCGAGAC
FGFR2C: fwd, TCTGAGGACTTTGTGAATGAC rev, TTTTCCTTTTCAGGAGCTGGC
FGFR3: fwd, ATTCAAGGGAGAGCACAGGA rev, ACTTGCTGCCGTTGACTTCT
INSR: fwd, AGACAGTGAGCTTTATGAACTC rev, ATGGAGCCCAGGTCTCTTCTCT
IGFR1: fwd, CTCAAAAGTTACCTGAGATCATTGA rev, TTGTGAAGACTCCATCCTTCAGTGA
IGFR2: fwd, TCATCTCTGACACGGCACAGAGAGAAC rev, CATCACACTTAAGGCGCAATATTGTTG
ALK2: fwd, CTCATCGGGACTTGAAGAGC rev, TAACCATGCGCCTAGCTACC
ALK3: fwd, ATGGGCATTGCTTTGCTATC rev, CAGAACTTTGCGACTGGTCA
ALK4: fwd, AGGTTTGCCACTTTTTGTGC rev, ACGTGCCATTCTTCTTCACC
ALK5: fwd, TTTGTGCACCATCCTTCAGA rev, CCATTTCCCCCTCCATACTT
ALK6: fwd, TTCTGGTGGACATTTGGTCA rev, ACTTCCCCATAGCGACCTTT
ALK7: fwd, GCTCCCTGTTCGACTACCTG rev, TGTTCATTGCGTCATCCAGT
ACVR2A: fwd, ACACAGCCAACTTCCAATCC rev, GGAAACCACGTTAGCCTTGA
ACVR2B: fwd, CTGTCAGTGGCCATCCTCTT rev, AGCTCGTTCCAGCTGATGAT
BMPR2: fwd, AAACCTGCAATATCCCATCG rev, TTTTCCCTGGACACCAAGAC
TGFβR2: fwd, CGCCCTAAAACCCCTATTGT rev, AGGGATGCTCTCGCACTTTA

Supplemental References

Glover, J.D., Taylor, L., Sherman, A., Zeiger-Poli, C., Sang, H.M., and McGrew, M.J. (2013). A novel piggyBac transposon inducible expression system identifies a role for AKT signalling in primordial germ cell migration. Plos One *8*, e77222.

Le Dréau, G., Garcia-Campmany, L., Rabadán, M.A., Ferronha, T., Tozer, S., Briscoe, J., and Martí, E. (2012). Canonical BMP7 activity is required for the generation of discrete neuronal populations in the dorsal spinal cord. Development *139*, 259–268.

Macdonald, J., Taylor, L., Sherman, A., Kawakami, K., Takahashi, Y., Sang, H.M., and McGrew, M.J. (2012). Efficient genetic modification and germ-line transmission of primordial germ cells using piggyBac and Tol2 transposons. P Natl Acad Sci USA *109*, E1466-E1472.

Mansour, S.J., Matten, W.T., Hermann, A.S., Candia, J.M., Rong, S., Fukasawa, K., Vande Woude, G.F., and Ahn, N.G. (1994). Transformation of mammalian cells by constitutively active MAP kinase kinase. Science *265*, 966–970.
